# The Role of Blood Pressure in Carotid Plaque Incidence: Interactions With Body Mass Index, Age, and Sex-Based on a 7-Years Cohort Study

**DOI:** 10.3389/fphys.2021.690094

**Published:** 2021-08-23

**Authors:** Jian Liu, Xuehua Ma, Xue-Ling Ren, Hong Xiao, Leyuan Yan, Zhuorong Li, Shengshu Wang

**Affiliations:** ^1^Department of Healthcare, Fourth Medical Center of Chinese PLA General Hospital, Beijing, China; ^2^Department of Respiratory, National Clinical Research Center for Geriatrics Diseases, Second Medical Center of Chinese PLA General Hospital, Beijing, China; ^3^Department of Healthcare, Central Military Commission, Beijing, China; ^4^Institute of Geriatrics, Beijing Key Laboratory of Aging and Geriatrics, National Clinical Research Center for Geriatrics Diseases, Second Medical Center of Chinese PLA General Hospital, Beijing, China

**Keywords:** carotid arteries, blood pressure, interaction, cohort studies, age

## Abstract

**Background:** Although high blood pressure (BP) is a risk factor for carotid plaque, its long-term prognostic value might be underestimated due to its confounding interactions with BMI, age, and gender. Therefore, we conducted a 7-year prospective cohort study to evaluate the prognostic value of BP for the incidence of carotid plaque.

**Methods:** The subjects enrolled in 2011 were free of carotid plaque at baseline and were followed up in 2018. Multivariate Cox proportional-hazards models were used to evaluate the association between BP and carotid plaque incidence.

**Results:** During the follow-up study, the incidence of carotid plaque was 36.5%. The significant positive linear trend showed that subjects with higher BP levels at baseline were more likely to develop carotid plaques at the end. Especially in the female subpopulation, after confounders being adjusted, the carotid plaque was associated with higher BP (adjusted HR 1.52, 95% CI 1.02–2.26), pulse pressure (PP) (adjusted HR 1.15, 95% CI 0.76–1.75), and mean arterial pressure (MAP) (adjusted HR 1.44, 95% CI 1.00–2.08). The adjusted HRs of hypertension, PP, and MAP (HR 27.71, 95% CI 2.27–338.64; HR 14.47, 95% CI 1.53–137.18; HR 9.97, 95% CI 1.29–77.28) were significantly higher after the potential antagonistic interactions between BP categorical indicators and age being adjusted, respectively.

**Conclusion:** High BP indicators might be associated with higher HRs of carotid plaque after adjusting interactions between BP indicators and BMI, age, and gender, which suggests that the incidence of carotid plaque in female adults with high BP indicators might increase significantly with the increase of age.

## Introduction

Approximately one-third of the adult population in China has been diagnosed with carotid atherosclerosis, leading to a heavy economic and social burden (Clarke et al., 2017). Prevention of carotid plaque is crucial because carotid plaque is associated with an increased risk of cardiovascular disease (Naqvi and Lee, 2014; Franceschini et al., 2018; Sillesen et al., 2018). The risk factors for carotid plaque include age, sex, hypertension, diabetes, and dyslipidemia (Noflatscher et al., 2018; Zhao and Hatsukami, 2018).

Hypertension is the leading modifiable risk factor for cardiovascular diseases including carotid plaque (Hellings et al., 2010; Franceschini et al., 2018; Sillesen et al., 2018). Inadequate awareness, improper therapeutic and poor controlling of hypertension might increase the risk of cardiovascular diseases (Zhao and Hatsukami, 2018). In a series of cohort studies and relatively small clinical trials studies, hypertension has been proposed as a risk factor for carotid plaque (Clarke et al., 2017; Steffen et al., 2018; Wang et al., 2018; Zhao and Hatsukami, 2018; GBD 2013 Mortality and Causes of Death, 2015). However, applying a single indicator of blood pressure (BP) to assess the risk of carotid plaque limits the ability in the evaluation of the prediction efficiency of different BP indicators, such as pulse pressure (PP), mean arterial pressure (MAP), and isolated systolic hypertension. Furthermore, the interactions between BP indicators and other cardiovascular risk factors for the carotid plaque have not been systematically analyzed (Yang et al., 2013; Clarke et al., 2017). Furthermore, this interaction may be involved in the formation of carotid plaque (Rovella et al., 2018) and the observational studies of these potential interactions were still unclear.

In this 7-year follow-up cohort study, we aimed to explore the possible interactions between BP categorical indicators and age, BMI, and gender in the associations of BP and incidence of carotid plaque.

## Materials and Methods

### Subjects

**Figure 1:**
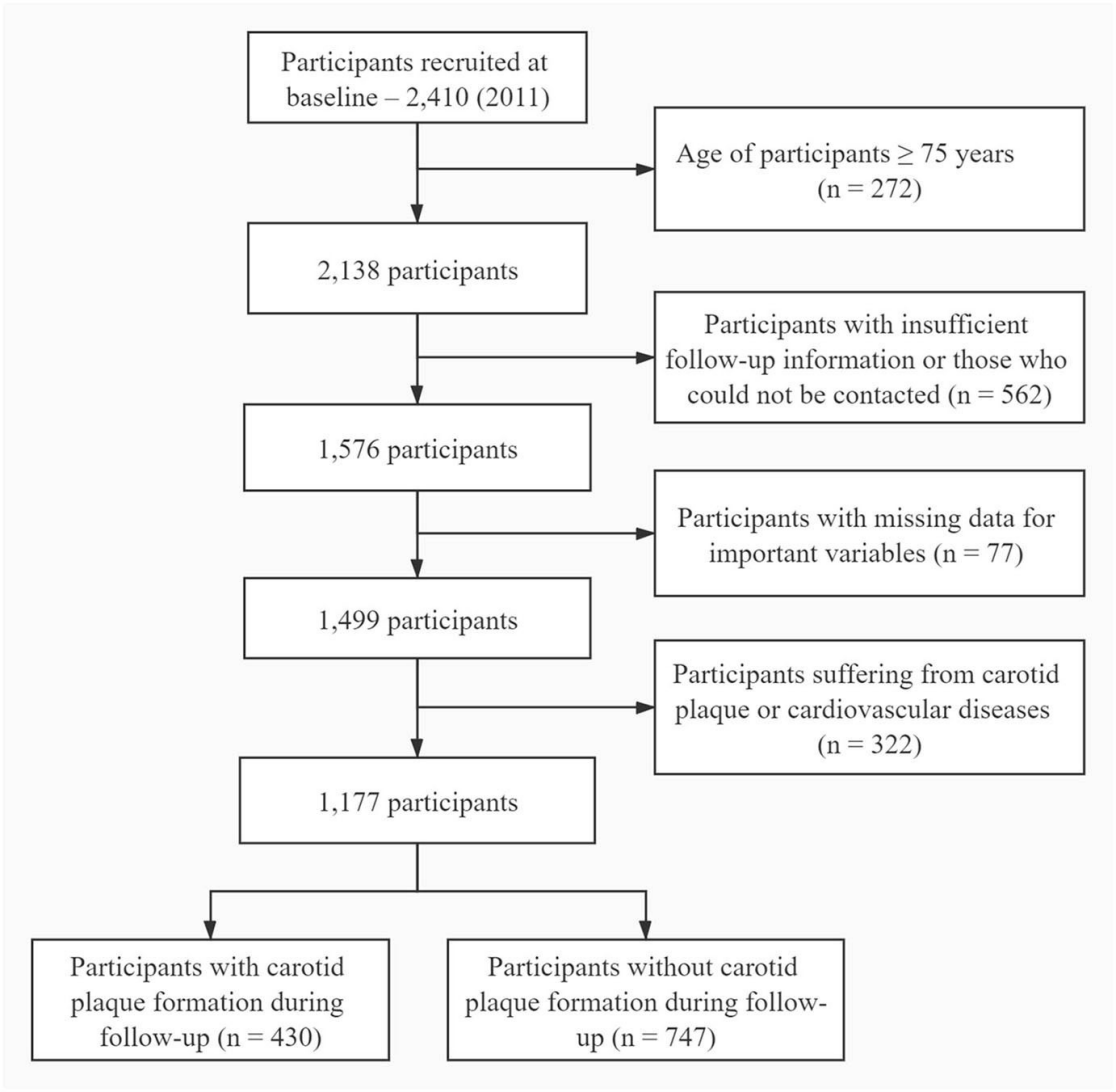
Flowchart depicting participant enrollment. Subjects who were between 35 and 74 years of age and had a full follow-up information were eligible for enrollment in the study. Participant aged 75 years or older were excluded (*n* = 272). All subjects who had insufficient follow-up information (*n* = 562), missing data (*n* = 77), or suffering from cardiovascular diseases or carotid plaque (*n* = 322) at baseline were also excluded.

A total of 2,410 subjects who were free of carotid plaque, coronary heart disease, stroke, or heart failure at baseline were recruited from the Department of Healthcare of the Fourth Medical Center of the People's Liberation Army General Hospital. The age of the study population was ranging from 35 to 74 years. Subjects with insufficient follow-up information and those who could not be contacted were excluded from the study. The baseline survey was conducted at 2011 with annual follow-up during physical examination, and the follow-up endpoint in this study was at 2018. Subjects lost to follow-up and those for whom relevant data were missing were not included in the data analyses (Figure 1). All subjects provided the written informed consent upon enrollment to the study. This study was approved by the ethics committee of Fourth Medical Center of the People's Liberation Army General Hospital.

### Measurement of Carotid Plaque

Carotid artery ultrasonography was performed using a B-mode ultrasound system (EPIQ7 or HD7XE, Philips, Amsterdam, Netherlands) with a linear array transducer (7.5–12 MHz). The examination included bilateral longitudinal and transverse scans of the distal segments of the common carotid artery, the bifurcation segments of the common carotid artery, and the proximal segments of the internal and external carotid arteries. We measured the intima-media thickness (IMT) and plaque formation in the wall of the eight segments of vessels mentioned above. The presence of plaque was defined as one of the following: IMT ≥ 1.5 mm, a focal structure (≥0.5 mm) protruding into the arterial lumen, or the surrounding IMT ≥ 50% (Collaborators, 2017; Dong et al., 2019).

Carotid plaque was defined as the development of at least one plaque in the eight previously plaque-free arterial segments during reexamination in 2018. To test for inter-observer reliability, plaque measurements in 25 patients were repeated after 1 week by two independent technicians and the results showed high reliability (intraclass correlation coefficients were 0.94).

### Covariants

Subjects were given a physical examination in the medical insurance hospital in May every year. The subjects were at a fasting state in the morning and BP was measured twice with a 3-mine interval by trained nurses using digital automatic monitors (HBP-1300; Omron, Netherlands), and an average value was calculated. Subjects with systolic blood pressure (SBP) <120 mmHg and diastolic blood pressure (DBP) <80 mmHg were defined as normal BP, while those with SBP of 120–139 mmHg and DBP of 80–89 mmHg were defined as high-normal BP. Subjects were classified as hypertensive if they had SBP ≥140 mmHg and/or DBP ≥90 mmHg (China Hypertension Prevention Control Guidelines Revision Committee, 2019).

To better understand the role of BP in carotid plaque incidence, we analyzed a range of BP categorical indicators, including hypertension, PP, isolated systolic hypertension, and MAP. Hypertension was defined as SBP >140 mmHg and/or DBP >90 mmHg according to an average BP of two measurements. Pre-hypertension was defined as SBP of 120–139 mmHg or DBP of 80–89 mmHg and normotensive subjects as SBP <120 mmHg or DBP <80 mmHg. Isolated systolic hypertension was defined as SBP ≥140 mmHg and DBP <90 mmHg. PP was categorized into three subgroups according to the tertile points such as T1 (≤38 mmHg), T2 (38 mmHg−47 mmHg), and T3 (>47 mmHg), and MAP were divided into tertiles such as ≤86 mmHg, 86–94 mmHg, and >94 mmHg.

Fasting venous blood samples were drawn and assayed for glucose, total cholesterol (TC), triglyceride (TG), low-density lipoprotein cholesterol (LDL-C), high-density lipoprotein cholesterol (HDL-C), and other biochemical parameters using an automatic analyzer (COBAS-701, Roche, Mannheim, Germany). Body mass index (BMI) was calculated as weight (kg) divided by height squared (m^2^). Individuals were classified as underweight (BMI <18.5 kg/m^2^), normal (BMI 18.5–23.9 kg/m^2^), or overweight (BMI ≥24.0 kg/m^2^) based on the BMI limits defined for Chinese adults (Department of Disease Control, Ministry of Health of the People's Republic of China, 2006). According to their fasting plasma glucose (FPG) levels, subjects were classified as normal FPG (≤99.0 mg/dl), high-normal FPG (99.1–108.2 mg/dl), impaired FPG (108.3–125.9 mg/dl), or diabetes (≥126.0 mg/dl) (Ding et al., 2015).

### Statistical Analyses

All data analyses were conducted with SPSS 24.0 (IBM, Armonk, NY, USA). Continuous data were presented as median and interquartile range (IQR), while categorical data as the number of cases and percentage (%). The normality of continuous variables was tested using the Shapiro–Wilk normality test. Inter-group differences in skewed data, such as sex-related variables, were assessed for significance using the Kruskal–Wallis test. Differences in categorical variables were assessed using the chi-squared test and *p* < 0.05 was considered statistically significant.

Multivariate Cox proportional-hazards models were built using the “enter” method for variable selection (Ding et al., 2015). In Model 1, age, dyslipidemia, FPG category, and BMI category were adjusted. Model 2 was adjusted for the same variables in Model 1 and the interaction between age and BP categorical indicators. Model 3 was adjusted for the same variables as Model 1 and the interaction between BMI and hypertension. *E*-value was reported in the sensitivity analysis, which is related to the potentially subject to unmeasured confounding (VanderWeele and Ding, 2017).

## Results

### Baseline Characteristics of Subjects

A total of 2,410 subjects were recruited in 2011, and the last follow-up was conducted in 2018 including 1,177 subjects (555 men and 622 women), of which 430 new cases of carotid plaque were found. The median age of the subjects was 51 years (IQR 41–61) ranging from 35 to 74 years. The proportion of women was slightly higher than that of men (52.85% vs. 47.15%). Abdominal circumference is 87 (82–93) in men vs. 86 cm (80–92 cm) in women. BMI (18.5–24.0 kg/m^2^) is 44.6% in men vs. 31.6% in women. DBP level is 75 mmHg (71–82 mmHg) in men vs. 74 mmHg (69–79 mmHg) in women (Table 1).

**Table 1:** Baseline characteristics of subjects.

	**All (***n*** = 1177)**	**Men (***n*** = 555)**	**Women (***n*** = 622)**	***p***
Age (year), median (IQR)	51.00 (41.00–61.00)	49.00 (40.00–58.50)	53.00 (43.00–62.00)	<0.001
Abdominal circumference (cm), median (IQR)	87.00 (81.00–93.00)	87.00 (82.00–93.00)	86.00 (80.00–92.00)	<0.001
SBP (mmHg), median (IQR)	118.00 (109.00–129.00)	119.00 (110.00–128.00)	118.00 (107.00–129.00)	0.429
DBP (mmHg), *median* (*IQR*)	75.00 (70.00–80.00)	75.00 (71.00–82.00)	74.00 (69.00–79.00)	<0.001
Marital status [*n* (%)]				<0.001
Married	858 (72.9%)	244 (43.9%)	614 (98.7%)	
Widowed/divorced	319 (27.1%)	311 (56.0%)	8 (1.2%)	
TC (mmol/L), median (IQR)	4.90 (4.32–5.49)	4.71 (4.14–5.21)	5.02 (4.49–5.66)	<0.001
TG (mmol/L), median (IQR)	1.14 (0.81–1.57)	1.24 (0.88–1.71)	1.06 (0.77–1.43)	<0.001
LDL-C (mmol/L), median (IQR)	2.94 (2.40–3.45)	2.86 (2.36–3.37)	2.98 (2.42–3.54)	0.012
HDL-C (mmol/L), median (IQR)	1.37 (1.18 −1.58)	1.25 (1.08–1.42)	1.50 (1.30–1.66)	<0.001
FPG (mg/dL), median (IQR)	95.40 (88.20–102.60)	95.40 (88.20–102.60)	93.6 (88.20–102.60)	0.327
BMI (kg/m^2^) [*n* (%)]				<0.001
<18.5	615 (52.2%)	245 (44.1%)	370 (59.4%)	
18.5–24.0	445 (37.8%)	248 (44.6%)	197 (31.6%)	
>24.0	117 (9.6%)	62 (11.1%)	55 (8.8%)	
Hypertension [*n* (%)]				<0.001
No	897 (76.2%)	465 (83.7%)	432 (69.4%)	
Yes	280 (23.7%)	90 (16.2%)	190 (30.5%)	
Dyslipidemia [*n* (%)]				0.756
No	847 (71.9%)	397 (71.5%)	450 (72.3%)	
Yes	330 (28.0%)	158 (28.4%)	172 (27.6%)	
FPG [*n* (%)]				0.944
Normal	786 (66.7%)	369 (66.4%)	417 (67.0%)	
High-normal	187 (15.8%)	91 (16.4%)	96 (15.4%)	
IFG	89 (7.5%)	40 (7.2%)	49 (7.8%)	
Diabetes	115 (9.7%)	55 (9.9%)	60 (9.6%)	

*Values are n (%) or median (IQR)*.

*SBP, systolic blood pressure; DBP, diastolic blood pressure; TC, total cholesterol; TG, triglyceride; LDL-C, low-density lipoprotein cholesterol; HDL-C, high-density lipoprotein cholesterol;*

*BMI, body mass index; FPG, fasting plasma glucose; IFG, impaired fasting glucose; IQR, interquartile range*.

### Incidence of Carotid Plaque and Association With Baseline BP

After analyzing 8,239 person-years of follow-up, we identified 430 cases of new-onset carotid plaque (36.5%; 95% CI 33.8–39.3%). The incidence was 5.22% per 100 person-years (95% CI 5.02–5.44%). The incidence of carotid plaque was significantly higher among male subjects and subjects whose baseline BP categorical indicators were higher (Table 2).

**Table 2:** Incidence of carotid atherosclerosis at 7-year follow-up in subjects stratified by baseline blood pressure (BP).

**Variable**	**Male carotid plaque (***n*** = 555)**				**Male carotid plaque (***n*** = 622)**			
	***n***	**Incidence (%, 95% CI)**	**Total person-years**	**Incidence rate per 100 person-years (%, 95% CI)**	**n**	**Incidence (%, 95% CI)**	**Total person-years**	**Incidence rate per 100 person-years (%, 95% CI)**
**Hypertension**								
No (*n* = 893)	159	17.8 (16.0–20.0)	6,251	2.5 (2.3–2.7)	104	11.6 (9.6–13.6)	6,251	1.7 (1.7–1.7)
Yes (*n* = 284)	52	18.3 (14.4–22.2)	1,988	2.6 (2.4–2.8)	115	40.5 (34.6–46.4)	1,988	5.8 (5.6–6.0)
**Blood pressure**								
Normal (*n* = 529)	77	14.5 (10.6–18.4)	3,703	2.1 (1.9–2.3)	47	8.9 (8.7–9.1)	3,703	1.3 (1.1–1.5)
High-normal (*n* = 364)	82	22.5 (18.6–26.4)	2,548	3.2 (3.1–3.4)	57	15.6 (11.7–19.5)	2,548	2.2 (2.0–2.4)
Hypertension (*n =* 284)	52	18.3 (14.4–22.2)	1,988	2.6 (2.4–2.8)	115	40.5 (34.6–46.4)	1,988	5.8 (5.6-6.0)
**Pulse pressure**								
T1 (*n =* 415)	52	12.5 (8.6–16.4)	2,905	1.8 (1.6–2.0)	42	10.1 (8.1–12.1)	2,905	1.4 (1.2–1.6)
T2 (*n =* 394)	79	20.1 (16.2–24.0)	2,758	2.9 (2.7–3.1)	55	14.0 (10.1–17.9)	2,758	2.0 (1.8–2.2)
T3 (*n =* 368)	80	21.7 (17.8–25.6)	2,576	3.1 (2.9–3.3)	122	33.2 (29.3–37.1)	2,576	4.7 (4.5–4.9)
**Isolated systolic hypertension**								
No (*n =* 1086)	192	17.7 (15.6–19.7)	7,602	2.5 (2.5–2.5)	175	16.1 (14.1–18.1)	7,602	2.3 (2.3–2.3)
Yes (*n =* 91)	19	20.9 (13.1–28.7)	637	3.0 (2.6–3.4)	44	48.4 (38.6–58.2)	637	6.9 (6.5–7.3)
**Mean arterial pressure**								
T1 (*n =* 401)	53	13.2 (9.3–17.1)	2,807	1.9 (1.7–2.1)	49	12.2 (8.3–16.1)	2,807	1.7 (1.5–1.9)
T2 (*n =* 397)	66	16.6 (12.7–20.5)	2,779	2.4 (2.2–2.6)	72	18.1 (14.2–22.0)	2,779	2.6 (2.4–2.8)
T3 (*n =* 379)	92	24.3 (20.4–28.2)	2,653	3.5 (3.3–3.7)	98	25.9 (22.0–29.8)	2,653	3.7 (3.5–4.1)
**Total**	211	38.0 (34.1–41.9)	3,885	5.4 (5.2–5.4)	219	35.2 (31.3–39.1)	4,354	5.0 (4.8–5.2)

*CI, confidence interval; T1, in the first tertile; T2, in the second tertile; T3, in the third tertile*.

### Incidence of Carotid Plaque and Its Association With BP Levels

**Figure 2:**
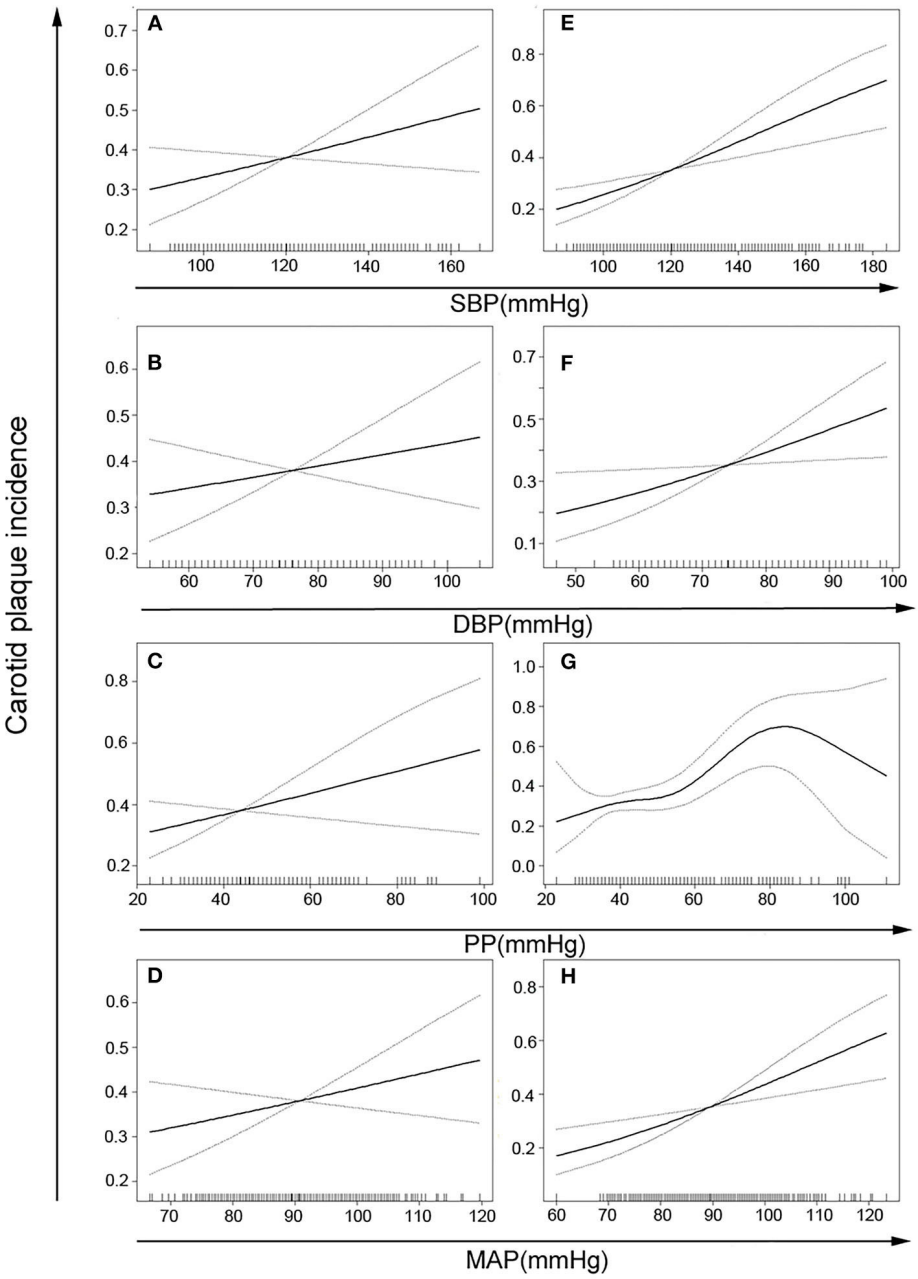
Age-adjusted risks of carotid plaque incidence based on different blood pressure indicators in women **(A–D)** or men **(E–H)**. There is a nonlinear correlation in panel G (*p* for nonlinear = 0.002) and positive linear correlation in other panels (*p* for trend <0.05). SBP, systolic blood pressure; DBP, diastolic blood pressure; PP, pulse pressure; MAP, mean arterial pressure.

In the analysis which takes BP (SBP, DBP, PP, and MAP) as continuous variables, the incidence of carotid plaque increased among male subjects with the increase of age-adjusted BP indicators. These associations were found in female subjects except the PP indicator (Figure 2).

### Incidence of Carotid Plaque and Association With BP Categorical Indicators

Multivariate Cox proportional hazards models were generated to calculate hazard ratios (HRs) of carotid plaque incidence in men and women according to different BP indicators, after adjusting for age, dyslipidemia, BMI, and FPG categories, and significant interactions between BP and age and BMI. Among women, the adjusted HRs for the carotid plaque were 8.80 (95% CI 1.46–52.98; Model 2) and 1.55 (95% CI 1.06–2.28; Model 3) in the presence of hypertension relative to normal or high-normal BP, while Model 1 indicated a much smaller HR (1.40, 95% CI 1.03–1.89). These adjusted HRs of women were higher than that of men (Table 3). Among women, the adjusted HRs for carotid plaque were 14.70 (95% CI 2.32–93.29; Model 2) in the presence of high-normal BP and 27.71 (95% CI 2.27–338.64; Model 2) in the presence of hypertension relative to normal BP. The adjusted HR for carotid plaque was 1.98 (95% CI 1.21–3.24; Model 3) in the presence of hypertension relative to normal BP. Model 1 indicated much smaller corresponding HRs (HR 1.34, 95% CI 0.96–1.87 and HR 1.52, 95% CI 1.02–2.26). Among men, the adjusted HRs in models 1–3 were not statistically significant (Table 4).

**Table 3:** Analysis of variables that may affect the association between hypertension and risk of carotid plaque.

	**Men**			**Women**		
**Variables**	**Model 1**	**Model 2**	**Model 3**	**Model 1**	**Model 2**	**Model 3**
	**HR (95% CI)**	**HR (95% CI)**	**HR (95% CI)**	**HR (95% CI)**	**HR (95% CI)**	**HR (95% CI)**
**Hypertension**						
No	1	1	1	1	1	1
Yes	1.10 (0.78–1.56)	0.88 (0.13–6.13)	1.05 (0.56–1.98)	1.40 (1.03–1.89)	8.80 (1.46–52.98)	1.55 (1.06–2.28)
**Age (year)**	1.04 (1.02–1.05)	1.04 (1.02–1.05)	1.04 (1.02–1.05)	1.06 (1.04–1.07)	1.07 (1.05–1.09)	1.06 (1.04–1.07)
**BMI (kg/m^2^)**						
<18.5	1	1	1	1	1	1
18.5–24.0	1.27 (0.95–1.71)	1.26 (0.94–1.70)	1.23 (0.89–1.72)	0.90 (0.66–1.22)	0.89 (0.66–1.21)	1.04 (0.68–1.60)
>24.0	1.15 (0.71–1.87)	1.15 (0.71–1.86)	1.24 (0.70–2.20)	1.32 (0.87–2.00)	1.22 (0.80–1.87)	1.50 (0.71–3.18)
**Dyslipidemia**						
No	1	1	1	1	1	1
Yes	1.01 (0.74–1.36)	0.94 (0.65–1.36)	1.00 (0.74–1.36)	1.14 (0.86–1.52)	1.11 (0.83–1.48)	1.15 (0.87–1.53)
**FPG**						
Normal	1	1	1	1	1	1
High-normal	0.85 (0.57–1.25)	0.85 (0.58–1.26)	0.84 (0.57–1.24)	0.99 (0.69–1.44)	0.99 (0.68–1.43)	1.00 (0.69–1.45)
IFG	0.96 (0.58–1.58)	0.97 (0.58–1.60)	0.95 (0.57–1.56)	0.92 (0.58–1.46)	0.94 (0.59–1.48)	0.92 (0.58–1.46)
Diabetes	1.13 (0.75–1.70)	1.15 (0.76–1.75)	1.14 (0.76–1.73)	1.05 (0.71–1.56)	1.06 (0.72–1.57)	1.06 (0.71–1.58)

*Model 1, adjusts for hypertension, age, dyslipidemia, BMI, and FPG categories. Model 2, adjusts for these variables and for the interaction between age and hypertension. Model 3, adjusts for the same variables as Model 1 and the interaction between BMI and hypertension*.

*BMI, body mass index; FPG, fasting plasma glucose; IFG, impaired fasting glucose; HR, hazard ratio; CI, confidence interval*.

**Table 4:** Analysis of variables that may affect the association between BP (normal, high-normal, and hypertension) and risk of carotid plaque.

	**Men**			**Women**		
**Variables**	**Model 1**	**Model 2**	**Model 3**	**Model 1**	**Model 2**	**Model 3**
	**HR (95% CI)**	**HR (95% CI)**	**HR (95% CI)**	**HR (95% CI)**	**HR (95% CI)**	**HR (95% CI)**
**Hypertension**						
Normal BP	1	1	1	1	1	1
High–normal BP	1.04 (0.76–1.41)	1.15 (0.29–4.62)	1.16 (0.84–16.17)	1.34 (0.96–1.87)	14.70 (2.32–93.29)	1.53 (0.93–2.54)
Hypertension	1.14 (0.72–1.80)	1.28 (0.13–12.75)	2.44 (0.80–73.84)	1.52 (1.02–2.26)	27.71 (2.27–338.64)	1.98 (1.21–3.24)
**Age (year)**	1.04 (1.02–1.05)	1.04 (1.02–1.06)	1.04 (1.02–1.05)	1.06 (1.04–1.07)	1.08 (1.06–1.11)	1.06 (1.02–1.07)
**BMI (kg/m^2^)**						
<18.5	1	1	1	1	1	1
18.5–24.0	1.27 (0.95–1.71)	1.27 (0.94–1.71)	1.29 (0.91–1.82)	0.94 (0.69–1.27)	0.89 (0.66–1.21)	1.21 (0.66–2.20)
>24.0	1.15 (0.71–1.87)	1.15 (0.71–1.87)	1.24 (0.63–2.45)	1.33 (0.87–2.01)	1.24 (0.81–1.90)	2.09 (0.28–15.40)
**Dyslipidemia**						
No	1	1	1	1	1	1
Yes	1.01 (0.75–1.37)	1.01 (0.75–1.39)	1.01 (0.75–1.37)	1.21 (0.91–1.60)	1.17 (0.89–1.55)	1.13 (0.851–1.51)
**FPG**
Normal	1	1	1	1	1	1
Upper range of normal	0.85 (0.58–1.26)	0.85 (0.58–1.26)	0.85 (0.57–1.25)	1.02 (0.70–1.47)	1.00 (0.69–1.46)	1.01 (0.70–1.47)
IFG	0.96 (0.58–1.58)	0.96 (0.58–1.59)	0.95 (0.58–1.58)	0.94 (0.59–1.48)	0.96 (0.61–1.51)	0.93 (0.59–1.47)
Diabetes	1.14 (0.76–1.72)	1.14 (0.76–1.72)	1.12 (0.75–1.69)	1.06 (0.72–1.57)	1.08 (0.73-1.59)	1.05 (0.71–1.57)

*Model 1, adjusting for hypertension, age, BMI, dyslipidemia, and FPG categories. Model 2, adjusting for hypertension, age, BMI, dyslipidemia, FPG categories, and interaction of age by hypertension. Model 3, adjusts for the same variables as Model 1 and the interaction between BMI and hypertension*.

*BMI, body mass index; BP, blood pressure; FPG, fasting plasma glucose; IFG, impaired fasting glucose; HR, hazard ratio; CI, confidence interval*.

We analyzed the incidence of carotid plaque according to PP tertiles. Among women, the adjusted HRs for subjects in the third tertile were 14.47 (95% CI 1.53–137.18; Model 2) and 1.83 (95% CI 1.01–3.32; Model 3), relative to the first tertile, and Model 1 gave a smaller HR of 1.15 (95% CI 0.76–1.75). Among men, the adjusted HRs for subjects in tertiles in Models 1–3 were not statistically significant and lower than that of women (Table 5).

**Table 5:** Analysis of variables that may affect the association between pulse pressure (PP) tertile and risk of carotid plaque.

**Variables**	**Men**			**Women**		
	**Model 1**	**Model 2**	**Model 3**	**Model 1**	**Model 2**	**Model 3**
	**HR (95% CI)**	**HR (95% CI)**	**HR (95% CI)**	**HR (95% CI)**	**HR (95% CI)**	**HR (95% CI)**
**PP**						
T1	1	1	1	1	1	1
T2	1.16 (0.82–1.66)	2.47 (0.48–12.85)	1.34 (0.76–2.36)	1.04 (0.68–1.58)	4.60 (0.42–50.56)	1.66 (0.90–3.04)
T3	1.13 (0.75–1.71)	2.47 (0.35–17.41)	1.25 (0.69–2.24)	1.15 (0.76–1.75)	14.47 (1.53–137.18)	1.83 (1.01–3.32)
**Age (year)**	1.04 (1.02–1.05)	1.05 (1.02–1.07)	1.04 (1.02–1.05)	1.06 (1.04–1.08)	1.09 (1.06–1.13)	1.06 (1.04–1.07)
**BMI (kg/m^2^)**						
<18.5	1	1	1	1	1	1
18.5–24.0	1.28 (0.95–1.71)	1.27 (0.95–1.71)	1.44 (0.81–2.57)	0.93 (0.69–1.26)	0.88 (0.65–1.21)	1.84 (0.96–3.55)
>24.0	1.16 (0.72–1.87)	1.16 (0.72–1.87)	1.58 (0.59–4.23)	1.43 (0.95–2.16)	1.38 (0.91–2.09)	2.89 (0.96–8.70)
**Dyslipidemia**						
No	1	1	1	1	1	1
Yes	1.02 (0.75–1.38)	1.02 (0.75–1.39)	1.01 (0.74–1.38)	1.21 (0.92–1.61)	1.20 (0.90–1.58)	1.20 (0.90–1.60)
**FPG**						
Normal	1	1	1	1	1	1
Upper range of normal	0.85 (0.57–1.25)	0.85 (0.58–1.26)	0.84 (0.57–1.24)	1.02 (0.70–1.48)	1.02 (0.71–1.49)	1.01 (0.70–1.48)
IFG	0.97 (0.58–1.59)	0.96 (0.58–1.59)	0.97 (0.59–1.60)	0.97 (0.61–1.52)	0.97 (0.61–1.52)	0.97 (0.61–1.52)
Diabetes	1.15 (0.77–1.73)	1.14 (0.76–1.71)	1.14 (0.76–1.72)	1.08 (0.73–1.61)	1.11 (0.75–1.65)	1.08 (0.73–1.60)

*Model 1, adjusting for PP tertiles, age, BMI, dyslipidemia, and FPG categories. Model 2, adjusting for PP tertiles, age, BMI, dyslipidemia, FPG categories, and interaction of age by PP tertiles. Model 3, adjusts for the same variables as Model 1 and the interaction between BMI and PP tertiles*.

*T1, in the first tertile; T2, in the second tertile; T3, in the third tertile*.

*BMI, body mass index; PP, pulse pressure; FPG, fasting plasma glucose; IFG, impaired fasting glucose; HR, hazard ratio; CI, confidence interval*.

When we analyzed the incidence according to MAP tertiles, among women, the adjusted HRs for subjects in the third tertile were 9.97 (95% CI 1.29–77.28; Model 2) and 2.05 (95% CI 1.25–3.35; Model 3), relative to the first tertile, and Model 1 gave a smaller HR of 1.44 (95% CI 1.00–2.08). Among men, the adjusted HRs for subjects in tertiles in Models 1–3 were not statistically significant and lower than that of women (Table 6). Isolated systolic hypertension was not significantly associated with carotid plaque after adjustment.

**Table 6:** Analysis of variables that may affect the association between mean arterial pressure (MAP) tertiles and risk of carotid plaque.

	**Men**			**Women**		
	**Model 1**	**Model 2**	**Model 3**	**Model 1**	**Model 2**	**Model 3**
	**HR (95% CI)**	**HR (95% CI)**	**HR (95% CI)**	**HR (95% CI)**	**HR (95% CI)**	**HR (95% CI)**
**MAP**						
T1	1	1	1	1	1	1
T2	0.99 (0.69–1.42)	0.64 (0.13–3.10)	1.22 (0.72–2.06)	1.17 (0.80–1.69)	4.16 (0.51–34.07)	1.39 (0.84–2.30)
T3	1.17 (0.82–1.67)	1.75 (0.37–8.42)	1.17 (0.67–2.02)	1.44 (1.00–2.08)	9.97 (1.29–77.28)	2.05 (1.25–3.35)
**Age (year)**	1.04 (1.02–1.05)	1.04 (1.01–1.06)	1.04 (1.02–1.05)	1.06 (1.05–1.07)	1.08 (1.05–1.11)	1.06 (1.04–1.07)
**BMI (kg/m^2^)**						
<18.5	1	1	1	1	1	1
18.5–24.0	1.26 (0.94–1.69)	1.27 (0.94–1.71)	1.52 (0.87–2.64)	0.92 (0.68–1.25)	0.89 (0.66–1.21)	1.44 (0.81–2.56)
>24.0	1.14 (0.70–1.84)	1.15 (0.70–1.86)	0.90 (0.21–3.83)	1.32 (0.87–2.01)	1.28 (0.84–1.96)	3.24 (0.44–24.13)
**Dyslipidemia**						
No	1	1	1	1	1	1
Yes	1.00 (0.74–1.36)	0.99 (0.73–1.35)	1.01 (0.74–1.37)	1.21 (0.91–1.60)	1.18 (0.89–1.56)	1.18 (0.89–1.57)
**FPG**						
Normal	1	1	1	1	1	1
Upper range of normal	0.85 (0.58–1.25)	0.86 (0.58–1.26)	0.84 (0.57–1.25)	1.02 (0.70–1.47)	0.99 (0.69–1.44)	1.02 (0.70–1.48)
IFG	0.97 (0.59–1.60)	0.96 (0.58–1.58)	0.96 (0.58–1.59)	0.93 (0.59–1.47)	0.94 (0.60–1.49)	0.94 (0.59–1.49)
Diabetes	1.14 (0.76–1.71)	1.13 (0.75–1.69)	1.14 (0.76–1.71)	1.09 (0.74–1.61)	1.09 (0.73–1.61)	1.09 (0.74–1.62)

*Model 1, adjusting for MAP tertiles, age, BMI, dyslipidemia, and FPG categories. Model 2, adjusting for MAP tertiles, age, BMI, dyslipidemia, FPG categories, and interaction of age by MAP tertiles. Model 3, adjusts for the same variables as Model 1 and the interaction between BMI and MAP tertiles. T1, the first tertile; T2, the second tertile; T3, the third tertile. BMI, body mass index; MAP, mean arterial pressure; FPG, fasting plasma glucose; IFG, impaired fasting glucose; HR, hazard ratio; CI, confidence interval*.

### Sensitivity Analysis

*E*-value was calculated to estimate the potential effects of unmeasured significant confounding factors on the carotid plaque. When *E*-value was more than two, considerable unmeasured significant confounding factors could be needed to negate the existing adjusted HRs. In the sensitivity analysis, after adjusted for interactions between BP and BMI and age, all the *E*-value were more than two, which indicated the current associations tended to be more stable.

## Discussion

This cohort study evaluated the associations between BP indicators and the incidence of carotid plaque in Chinese adults. The findings showed that higher BP indicators might be associated with higher HRs of carotid plaque after adjusting for interactions between BP indicators and parameters such as BMI, age, and gender. These associations were significant in female subjects.

It is well-established that age, obesity, and hypertension are independent risk factors for carotid plaque (Genuth et al., 2003; Mahmoudi et al., 2011; Schwartz et al., 2016; Colafella and Denton, 2018). It is clear that there is an association between hypertension and the incidence of carotid plaque, but few studies have found that whether interactions among hypertension and its important influencing factors such as age, BMI, gender, and also whether the interactions could increase their influence on the onset of carotid plaque (Rovella et al., 2018). In this study, we observed that interactions of BP indicators with age or BMI were associated with the carotid plaque incidence in female adults. After the confounding factors and interactions of BP indicators with age and BMI being adjusted, high BP indicators significantly elevated the HRs of carotid plaque, while the HRs were lower if we adjusted only for confounding factors. Results were similar when we estimated the influence of PP and MAP tertiles on the incidence of carotid plaque. This suggests that age and BMI may act as both confounders and effect modifiers to influence the HRs of carotid plaque in female population. In addition, obesity was found to interact with age, sex, and BP on the incidence of carotid plaque, and the risk of carotid plaque due to overweight or obesity after adjusting for these interactions was substantially higher than that of when interactions were not controlled (Clarke et al., 2017). This might suggest that, after interactions being adjusted, the effects of high BP indicators on the incidence of carotid plaque might be weakened.

High BP indicators may induce carotid plaque *via* several potential mechanisms, including hereditary processes, endothelial dysfunction, and neurohumoral, humoral, and metabolic pathways (Chen et al., 2015). High BP level is accompanied by changes in the sympathetic nervous system, which may eventually lead to the conditioning of cardiovascular responses (Touboul et al., 2012). Endothelial dysfunction caused by hypertension leads to thrombosis and vascular occlusion, contributing to the formation of atherosclerotic plaque (Mancia et al., 2013; Joint Committee for Guideline R, 2019). Genome-wide association studies have identified a number of genetic susceptibility variants associated with the incidence of carotid atherosclerosis (Joint Committee for Guideline, R., 2006; Chen et al., 2015). In brief, higher BP levels leading to carotid plaque are generally known. Moreover, the combined mechanism of BP and influencing factors for the cardiac disease was also identified through the metabolic pathway and gene inheritance (Genuth et al., 2003; Clarke et al., 2017).

In this study, we conducted stratified analysis according to gender, and the significant gender differences in the associations between BP indicators and carotid plaque incidence were found. This could be due to sex differences in the activation of the sympathetic nervous system, renin-angiotensin-aldosterone system, and immune system. These systems play an important role in the effect of hypertension on carotid plaque (Schwartz et al., 2016). Another potential explanation is gender differences in the sex hormones estrogen and testosterone and in the sex chromosome complement (Schwartz et al., 2016). In addition, it could be due to the sex differences in lifestyle. Unhealthy lifestyle habits such as smoking, drinking, and staying up late are typically more common in men (Liu and Li, 2015; Rosendorff et al., 2015). The incidence of carotid plaque might be attributed to the fact that the effect of an unhealthy lifestyle on the carotid plaque was more than that of BP indicators. Future work should explore some of these hypotheses in detail to clarify the apparent sex differences in how BP influences the incidence of carotid plaque.

There is a significant difference in BP changes both in men and women in the whole life cycle. Among middle-aged and elderly women, due to hormone levels, obesity and aging, the average BP level increased faster, and they were more likely to suffer from hypertension. In addition, women might be more likely to be obese during and after menopause. Moreover, obesity increases BP in men and women, but women have higher BP. Therefore, middle-aged and elderly women are more likely to suffer from obesity, hypertension, and the combined effects of these factors multiply increase the risk of cardiovascular diseases (Ventura-Clapier et al., 2017; Colafella and Denton, 2018).

This study is a 7-year follow-up with a large sample size, which allowed us to analyze 430 cases of new-onset carotid plaque. It is of great significance to explore the influencing factors of carotid plaques formation since it can provide important evidence for the primary prevention of cardiovascular diseases. Another strength is that the diagnosis of carotid plaque was conducted using color ultrasonography to detect IMT of the bilateral carotid arteries at eight locations. Meanwhile, we proposed that high BP significantly increases the HRs of carotid plaque after adjusting for covariants and interactions between BP indicators and parameters such as BMI, age, and gender. The influence of hypertension on carotid plaque formation depends on age, sex, and BMI to some extent. Clinicians should pay more attention to BP management of elder and fatter women to prevent carotid plaque.

Limitations of this study are the lack of information on BP changes and follow-up. Therefore, this result should be interpreted with caution since baseline BP cannot capture fluctuations in BP during follow-up, so we were unable to explore the effects of ambulatory BP on the incidence of carotid plaque. In addition, a lack of information on the occurrence of carotid plaque during a 7-year follow-up may cause some bias. Moreover, we did not collect complete data on the use of antihypertensive or other drugs for cardiovascular disease, or on lifestyle factors that might influence the incidence such as smoking, drinking, and physical activity.

## Conclusion

In this study, we find that high BP indicators might be associated with higher HRs of carotid plaque after the interactions being adjusted in female adults, suggesting that the incidence of carotid plaque in female adults with high BP indicators and higher age or BMI might significantly increase. This hypothesis, however, needs further verification.

## Data Availability Statement

The raw data supporting the conclusions of this article will be made available by the authors, without undue reservation.

## Ethics Statement

The studies involving human participants were reviewed and approved by Ethics Committee of the fourth medical center of PLA General Hospital. The patients/participants provided their written informed consent to participate in this study. Written informed consent was obtained from the individual(s) for the publication of any potentially identifiable images or data included in this article.

## Author Contributions

JL and SW conceived the study and its design. JL, XM, X-LR, LY, HX, and ZL collected the data. JL, SW, XM, and X-LR managed, analyzed, and interpreted the data. All the authors have read and approved the final manuscript.

## Conflict of Interest

The authors declare that the research was conducted in the absence of any commercial or financial relationships that could be construed as a potential conflict of interest.

## Publisher's Note

All claims expressed in this article are solely those of the authors and do not necessarily represent those of their affiliated organizations, or those of the publisher, the editors and the reviewers. Any product that may be evaluated in this article, or claim that may be made by its manufacturer, is not guaranteed or endorsed by the publisher.

## References

[B1] Chen Y. Xiong H. Wu D. Pirbhulal S. Tian X. Zhang R.. (2015). Relationship of short-term blood pressure variability with carotid intima-media thickness in hypertensive patients. Biomed. Eng. Online 14:71. 10.1186/s12938-015-0059-8 26204889 PMC4511984

[B2] China Hypertension Prevention and Control Guidelines Revision Committee (2019). Guidelines for the prevention and treatment of hypertension in China. Chin. J. Cardiol. Med. 24, 24–56. 10.3969/j.issn.1007-5410.2019.01.002

[B3] Clarke R. Du H. Kurmi O. Parish S. Yang M. Arnold M.. (2017). Burden of carotid artery atherosclerosis in Chinese adults: implications for future risk of cardiovascular diseases. Eur. J. Prev. Cardiol. 24, 647–656. 10.1177/2047487317689973 28128654 PMC6675599

[B4] Colafella K. M. M. Denton K. M. (2018). Sex-specific differences in hypertension and associated cardiovascular disease. Nat. Rev. Nephrol. 14, 185–201. 10.1038/nrneph.2017.189 29380817

[B5] Collaborators G. B. D. M. (2017). Global, regional, and national under-5 mortality, adult mortality, age-specific mortality, and life expectancy, 1970–2016: a systematic analysis for the Global Burden of Disease Study 2016. Lancet 390, 1084–1150. 10.1016/S0140-6736(17)31833-0 28919115 PMC5605514

[B6] Department of Disease Control Ministry of Health of the People's Republic of China. (2006). The Guideline for Prevention and Control of Overweight and Obesity in Chinese Adults. Beijing: People's Medical Publishing House.

[B7] Ding J. Mitchell G. F. Bots M. L. Sigurdsson S. Harris T. B. Garcia M.. (2015). Carotid arterial stiffness and risk of incident cerebral microbleeds in older people: the Age, Gene/Environment Susceptibility (AGES)-Reykjavik study. Arterioscler. Thromb. Vasc. Biol. 35, 1889–1895. 10.1161/ATVBAHA.115.305451 26112009 PMC4514556

[B8] Dong S. Gao J. Wang C. Liu J. Gu H. Tu J.. (2019). Association between blood pressure components and the presence of carotid plaque among adults aged 45 years and older: a population-based cross-sectional study in rural China. Blood Press. Monit. 24, 234–240. 10.1097/MBP.0000000000000396 31469693

[B9] Franceschini N. Giambartolomei C. de Vries P. S. Finan C. Bis J. C. Huntley R. P.. (2018). GWAS and colocalization analyses implicate carotid intima-media thickness and carotid plaque loci in cardiovascular outcomes. Nat. Commun. 9:5141. 10.1038/s41467-018-07340-5 30510157 PMC6277418

[B10] Genuth S. Alberti K. G. Bennett P. Buse J. Defronzo R. Kahn R.. (2003). Follow-up report on the diagnosis of diabetes mellitus. Diabetes Care 26, 3160–3167. 10.2337/diacare.26.11.3160 14578255

[B11] Hellings W. E. Peeters W. Moll F. L. Piers S. R. van Setten J. Van der Spek P. J.. (2010). Composition of carotid atherosclerotic plaque is associated with cardiovascular outcome: a prognostic study. Circulation 121, 1941–1950. 10.1161/CIRCULATIONAHA.109.887497 20404256

[B12] Joint Committee for Guideline R (2019). 2018 Chinese guidelines for prevention and treatment of hypertension—a report of the revision committee of Chinese guidelines for prevention and treatment of hypertension. J. Geriatr. Cardiol. 16, 182–241. 10.11909/j.issn.1671-5411.2019.03.014 31080465 PMC6500570

[B13] Liu H. H. Li J. J. (2015). Aging and dyslipidemia: a review of potential mechanisms. Ageing Res. Rev. 19, 43–52. 10.1016/j.arr.2014.12.001 25500366

[B14] Mahmoudi M. Hill P. C. Xue Z. Torguson R. Ali G. Boyce S. W.. (2011). Patients with severe asymptomatic carotid artery stenosis do not have a higher risk of stroke and mortality after coronary artery bypass surgery. Stroke 42, 2801–2805. 10.1161/STROKEAHA.111.618082 21817149

[B15] Mancia G. Fagard R. Narkiewicz K. Redon J. Zanchetti A. Bohm M.. (2013). 2013 ESH/ESC guidelines for the management of arterial hypertension: the Task Force for the Management of Arterial Hypertension of the European Society of Hypertension (ESH) and of the European Society of Cardiology (ESC). Eur. Heart J. 34, 2159–2219. 10.1093/eurheartj/eht151 23771844

[B16] Mortality G. B. D. Causes of Death C. (2015). Global, regional, and national age-sex specific all-cause and cause-specific mortality for 240 causes of death, 1990–2013: a systematic analysis for the Global Burden of Disease Study 2013. Lancet 385, 117–171. 10.1016/S0140-6736(14)61682-2 25530442 PMC4340604

[B17] Naqvi T. Z. Lee M. S. (2014). Carotid intima-media thickness and plaque in cardiovascular risk assessment. JACC Cardiovasc. Imaging 7, 1025–1038. 10.1016/j.jcmg.2013.11.014 25051948

[B18] Noflatscher M. Schreinlechner M. Sommer P. Kerschbaum J. Berggren K. Theurl M.. (2018). Influence of traditional cardiovascular risk factors on carotid and femoral atherosclerotic plaque volume as measured by three-dimensional ultrasound. J. Clin. Med. 8:32. 10.3390/jcm8010032 30602707 PMC6352255

[B19] Rosendorff C. Lackland D. T. Allison M. Aronow W. S. Black H. R. Blumenthal R. S.. (2015). Treatment of hypertension in patients with coronary artery disease: a scientific statement from the American Heart Association, American College of Cardiology, and American Society of Hypertension. Hypertension 65, 1372–1407. 10.1161/HYP.0000000000000018 25828847

[B20] Rovella V. Anemona L. Cardellini M. Scimeca M. Saggini A. Santeusanio G.. (2018). The role of obesity in carotid plaque instability: interaction with age, gender, and cardiovascular risk factors. Cardiovasc. Diabetol. 17:46. 10.1186/s12933-018-0685-0 29598820 PMC5874994

[B21] Schwartz J. E. Burg M. M. Shimbo D. Broderick J. E. Stone A. A. Ishikawa J.. (2016). Clinic blood pressure underestimates ambulatory blood pressure in an untreated employer-based US population: results from the masked hypertension study. Circulation 134, 1794–1807. 10.1161/CIRCULATIONAHA.116.023404 27920072 PMC5151173

[B22] Sillesen H. Sartori S. Sandholt B. Baber U. Mehran R. Fuster V. (2018). Carotid plaque thickness and carotid plaque burden predict future cardiovascular events in asymptomatic adult Americans. Eur. Heart J. Cardiovasc. Imaging 19, 1042–1050. 10.1093/ehjci/jex239 29059296

[B23] Steffen B. T. Guan W. Stein J. H. Tattersall M. C. Kaufman J. D. Sandfort V.. (2018). Plasma *n*-3 and *n*-6 fatty acids are differentially related to carotid plaque and its progression: the multi-ethnic study of atherosclerosis. Arterioscler. Thromb. Vasc. Biol. 38, 653–659. 10.1161/ATVBAHA.117.310366 29326315 PMC5823763

[B24] Touboul P. J. Hennerici M. G. Meairs S. Adams H. Amarenco P. Bornstein N.. (2012). Mannheim carotid intima-media thickness and plaque consensus (2004–2006–2011). An update on behalf of the advisory board of the 3rd, 4th and 5th watching the risk symposia, at the 13th, 15th and 20th European Stroke Conferences, Mannheim, Germany, 2004, Brussels, Belgium, 2006, and Hamburg, Germany, 2011. Cerebrovasc. Dis. 34, 290–296. 10.1159/000343145 23128470 PMC3760791

[B25] VanderWeele T. J. Ding P. (2017). Sensitivity analysis in observational research: introducing the E-value. Ann. Intern. Med. 167, 268–274. 10.7326/M16-2607 28693043

[B26] Ventura-Clapier R. Dworatzek E. Seeland U. Kararigas G. Arnal J. F. Brunelleschi S.. (2017). Sex in basic research: concepts in the cardiovascular field. Cardiovasc. Res. 113, 711–724. 10.1093/cvr/cvx066 28472454

[B27] Wang Z. Chen Z. Zhang L. Wang X. Hao G. Zhang Z.. (2018). Status of hypertension in China: results from the China Hypertension Survey, 2012–2015. Circulation 137, 2344–2356. 10.1161/CIRCULATIONAHA.117.032380 29449338

[B28] Yang G. Wang Y. Zeng Y. Gao G. F. Liang X. Zhou M.. (2013). Rapid health transition in China, 1990–2010: findings from the Global Burden of Disease Study 2010. Lancet 381, 1987–2015. 10.1016/S0140-6736(13)61097-1 23746901 PMC7159289

[B29] Zhao X. Q. Hatsukami T. S. (2018). Risk factors for development of carotid plaque components. JACC Cardiovasc. Imaging 11(2 Pt 1), 193–195. 10.1016/j.jcmg.2016.12.027 28412422

